# Calcineurin Controls Cellular Prion Protein Expression in Mouse Astrocytes

**DOI:** 10.3390/cells11040609

**Published:** 2022-02-10

**Authors:** Giulia Dematteis, Elena Restelli, Virginia Vita Vanella, Marcello Manfredi, Emilio Marengo, Marco Corazzari, Armando A. Genazzani, Roberto Chiesa, Dmitry Lim, Laura Tapella

**Affiliations:** 1 Department of Pharmaceutical Sciences, Università del Piemonte Orientale “Amedeo Avogadro”, 28100 Novara, Italy; giulia.dematteis@uniupo.it (G.D.); armando.genazzani@uniupo.it (A.A.G.); 2 Department of Neuroscience, Istituto di Ricerche Farmacologiche Mario Negri IRCCS, 20156 Milan, Italy; elena.restelli@marionegri.it (E.R.); roberto.chiesa@marionegri.it (R.C.); 3 Department of Translational Medicine, Center for Translational Research on Autoimmune and Allergic Disease (CAAD), Università del Piemonte Orientale “Amedeo Avogadro”, 28100 Novara, Italy; virginia.vanella@uniupo.it (V.V.V.); marcello.manfredi@uniupo.it (M.M.); 4 Department of Sciences and Technological Innovation, Università del Piemonte Orientale “Amedeo Avogadro”, 28100 Novara, Italy; emilio.marengo@uniupo.it; 5 Department of Health Science (DSS), Center for Translational Research on Autoimmune and Allergic Disease (CAAD) & Interdisciplinary Research Center of Autoimmune Diseases (IRCAD), Università del Piemonte Orientale “Amedeo Avogadro”, 28100 Novara, Italy; marco.corazzari@uniupo.it

**Keywords:** astrocytes, calcineurin, FK506, prion protein, protein synthesis, calcium, neuroinflammation

## Abstract

Prion diseases arise from the conformational conversion of the cellular prion protein (PrP^C^) into a self-replicating prion isoform (PrP^Sc^). Although this process has been studied mostly in neurons, a growing body of evidence suggests that astrocytes express PrP^C^ and are able to replicate and accumulate PrP^Sc^. Currently, prion diseases remain incurable, while downregulation of PrP^C^ represents the most promising therapy due to the reduction of the substrate for prion conversion. Here we show that the astrocyte-specific genetic ablation or pharmacological inhibition of the calcium-activated phosphatase calcineurin (CaN) reduces PrP^C^ expression in astrocytes. Immunocytochemical analysis of cultured CaN-KO astrocytes and isolation of synaptosomal compartments from the hippocampi of astrocyte-specific CaN-KO (ACN-KO) mice suggest that PrP^C^ is downregulated both in vitro and in vivo. The downregulation occurs without affecting the glycosylation of PrP^C^ and without alteration of its proteasomal or lysosomal degradation. Direct assessment of the protein synthesis rate and shotgun mass spectrometry proteomics analysis suggest that the reduction of PrP^C^ is related to the impairment of global protein synthesis in CaN-KO astrocytes. When WT-PrP and PrP-D177N, a mouse homologue of a human mutation associated with the inherited prion disease fatal familial insomnia, were expressed in astrocytes, CaN-KO astrocytes showed an aberrant localization of both WT-PrP and PrP-D177N variants with predominant localization to the Golgi apparatus, suggesting that ablation of CaN affects both WT and mutant PrP proteins. These results provide new mechanistic details in relation to the regulation of PrP expression in astrocytes, suggesting the therapeutic potential of astroglial cells.

## 1. Introduction

Prion diseases are a group of neurodegenerative diseases affecting humans and animals characterized by the accumulation of a misfolded isoform of the cellular prion protein (PrP^C^), commonly referred to as PrP^Sc^, in the brain [1]. Human prion diseases include (I) iatrogenic forms, e.g., Kuru, iatrogenic Creutzfeldt–Jakob disease (iCJD) and variant CJD; (II) sporadic forms, e.g., sporadic CJD and fatal insomnia (sFI); and (III) familial forms carrying point or insertional mutations in the *PRNP* gene encoding PrP^C^, such as genetic CJD, fatal familial insomnia (FFI) and Gerstmann–Straussler–Scheinker (GSS) syndrome [2,3]. Prion diseases have a long incubation period before clinical manifestation. Patients develop heterogeneous symptoms, such as dementia, ataxia, myoclonus and other motor and neuropsychiatric manifestations, that lead to a fatal outcome. Spongiform vacuolation of the grey matter, neuronal loss and chronic inflammation, with astrogliosis and microgliosis, are the main neuropathological hallmarks of prion diseases [4].

In prion research, many different mouse and cellular models have been described to understand the pathological mechanism and to define a possible therapeutic strategy [5,6,7], with the main efforts focused on neurons. However, a growing body of evidence suggests that astroglial cells can contribute to the pathogenesis of prion diseases [8,9].

Astrocytes are the principal homeostatic cells of the central nervous system (CNS). They are responsible for structural, functional and metabolic support to neurons and other cells in the CNS [10,11,12]. In prion diseases, astrocytes become reactive, losing their ability to control the CNS environment [6,13]. Reactive gliosis is an early pathological marker of neuropathology, which develops in concomitance with prion deposition and leads to neuronal dysfunction [13]. It has been reported that the expression of PrP^C^ in astrocytes contributes to their differentiation and is necessary for correct neuronal function [14,15,16].

Calcium (Ca^2+^) signals are essential for cell life, and, if not properly controlled, aberrant Ca^2+^ signals may result in cell malfunction or even cell death [17,18]. To induce specific downstream signaling events, Ca^2+^ signals are decoded by specialized Ca^2+^-binding proteins, the most important of which is, perhaps, the Ca^2+^/calmodulin-activated phosphatase calcineurin (CaN). Structurally, CaN is a heterodimer, composed of a catalytic CaNA subunit and an obligatory regulatory CaNB subunit. Binding of Ca^2+^ ions to CaNB and concomitant interaction of the Ca^2+^/calmodulin complex with CaNA lead to displacement of the autoinhibitory domain, enabling the interaction of CaN with its substrates. In the brain, CaN is expressed at high levels in neurons, in which it plays a fundamental role during neuronal plasticity [17]. However, CaN expressed in astroglial cells receives continuously increasing attention due to its key role in processes of neuroinflammation and reactive astrogliosis, which are characteristics of virtually all chronic neuropathological conditions. CaN acts through both direct protein dephosphorylation and activation of gene transcription, e.g., through the direct activation of transcription factors of activated T-cells (NFAT) [10,19,20]. Over-activation of CaN plays a pivotal role in reactive gliosis and neuroinflammation in Alzheimer’s disease (AD) [10,11,20,21,22,23,24]. A preclinical alteration of CaN has been reported in the TgPG14 mouse model of inherited prion disease [25,26], and treatment of prion-infected mice with the CaN inhibitor FK506 delays disease onset and promotes PrP degradation [27,28,29]. Moreover, PrP expression level influences the incubation period and disease duration in both prion-infected and mutant PrP mice [30,31,32,33,34]. Recently, a physiological role for CaN in astrocytes has been proposed, correlated with the control of neuronal excitability and astrocytic protein synthesis [35,36,37].

A growing body of evidence suggests that reduction of PrP^C^ expression represents a promising strategy to mitigate the conversion of PrP^C^ into PrP^Sc^ [38,39,40,41,42,43]. Here we show that genetic or pharmacological ablation of CaN in astrocytes reduces PrP^C^ expression at a post-transcriptional level. This regulation occurs at the level of ribosomal protein synthesis and localization of PrP^C^ at the plasma membrane and does not involve PrP^C^ degradation. Altogether, our data suggest that astroglial CaN may represent a novel target for manipulation with PrP^C^ expression and mitigation of prion diseases.

## 2. Materials and Methods

### 2.1. Astrocyte-Specific CaN-KO Mice (ACN-KO)

The generation and handling of a mouse line with conditional CaN knockout (KO) in GFAP-expressing astrocytes has been described previously [36]. The mice were housed in the animal facility of the Università del Piemonte Orientale, with unlimited access to water and food. Animals were managed in accordance with European directive 2010/63/UE and with Italian law D.l. 26/2014. The procedures were approved by the local animal-health and ethical committee (Università del Piemonte Orientale) and were authorized by the national authority (Istituto Superiore di Sanità; authorization numbers N. 214-2019 and N. 1136-2020). All efforts were made to reduce the number of animals by following the 3Rs rule.

### 2.2. Primary Hippocampal Astrocytic and Neuronal Cultures

Primary astroglial cultures were obtained by extracting hippocampi from either control (ACN-Ctr) or ACN-KO mouse pups at postnatal day 1–5 (P1–P5). Hippocampi were dissected in cold HBSS from the pups’ brains and dissociated by incubation with trypsin (0.5 mg/mL, 37 °C, 20 min) followed by gentle trituration and resuspension in Dulbecco’s Modified Eagle’s Medium (DMEM, Sigma, St Louis, MO, USA, Cat. D5671)—high glucose, supplemented with 10% foetal bovine serum (FBS, Gibco, Thermo Fisher Scientific, Milan, Italy, Cat. 10270-106), 2 mg/mL glutamine (Sigma, MERK Life Sciences srl, Milan, Italy, Cat. G7513), 10 U/mL penicillin and 100 mg/mL streptomycin (Sigma, MERK Life Sciences srl, Milan, Italy, Cat. P0781). Each pup was processed separately and samples were plated on 6-well plates pre-treated with 0.1 mg/mL poly-L-lysine (PLL, Sigma, MERK Life Sciences srl, Milan, Italy, Cat. P2636). At sub-confluence (5–10 days in vitro), cells were detached with trypsin and pleated for experiments. Neuron cultures were prepared as described [35,36,44].

### 2.3. Pharmacological Treatments

At passage 2 (P2), astroglial primary cells were plated at 60% of confluence in PLL-coated 6-well plates. Treatment was started 24 h later using 200 nM FK506 (TOCRIS, biotecne, Minneapolis, MN, USA, Cat. 3631). Cells were lysed after 1 week of treatment. To inhibit proteasome activity, 3 h before lysis, cells were treated with 1 µM (R)-MG132 (TOCRIS, biotecne, Minneapolis, MN, USA, Cat. 6033). To inhibit lysosome activity, 1 h before lysis, cells were treated with 20 µM chloroquine (Sigma, MERK Life Sciences srl, Milan, Italy, C6628-25G).

### 2.4. Assessment of Protein Synthesis

The global protein synthesis rate was assessed using the surface sensing of translation (SUnSET) method published previously [35,45]. Briefly, primary astroglial cells were incubated with 4 µM puromycin dihydrochloride (Sigma, MERK Life Sciences srl, Milan, Italy, Cat. P8833) in complete DMEM at 37 °C with 5% CO_2_ for 3 h [45]. Subsequently, cell lysates were fixed for immune fluorescence (IF) analysis with an anti-puromycin antibody.

### 2.5. Immunofluorescence

Primary Ctr and CaN-KO astrocytes, grown on 13 mm glass coverslips, were treated as previously explained. IF was performed as follows.

Puromycin IF. Cells were fixed in 4% paraformaldehyde and 4% sucrose, permeabilized (7 min in 0.1% Triton X-100 in phosphate-buffered saline (PBS)), blocked in 0.1% gelatine and immunoprobed with an appropriate primary antibody overnight at 4 °C. After three washes in PBS, an Alexa-conjugated secondary antibody (1:200) was applied for 1 h at RT. The following primary antibodies were used: anti-puromycin [35]. Secondary antibodies were as follows: Alexa Fluor 488 anti-mouse IgG. Nuclei were counter-stained with 4′,6-diamidino-2-phenylindole (DAPI).

PrP IF. Astrocytes were fixed in 4% paraformaldehyde for 7 min at room temperature and incubated in blocking solution (0.05% saponin, 0.5% bovine serum albumin (BSA), 10% FBS and 50 mM NH_4_Cl in PBS) for 30 min at room temperature (RT). The cells were then incubated with the anti-PrP antibody 12B2 (kindly provided by Dr. J.P. Langeveld, Central Veterinary Institute of Wageningen University, Lelystad, The Netherlands, to Dr. Roberto Chiesa) and diluted 1:400 in blocking solution overnight at 4 °C. After three washes in PBS, cells were incubated with a biotinylated anti-IgG secondary antibody (Vector; 1:200) for 1 h at RT, then reacted with Alexa Fluor 488-conjugated streptavidin (Thermo Fisher Scientific, Milan, Italy; 1:500) for 30 min at RT.

ER and Golgi markers IF. After fixation and blocking, cells were incubated with the primary antibodies diluted in blocking solution for 2–3 h at RT, then washed three times in PBS and incubated with a fluorescent conjugated anti-IgG secondary antibody for 1 h at RT. The following antibodies were used: mouse monoclonal anti-GM130 and rabbit polyclonal anti-BAP31. Secondary antibodies were conjugated with Alexa-594 or Alexa-647 fluorophores (Thermo Fisher Scientific, Milan, Italy; 1:500). Primary antibodies were listed in Table 1.

### 2.6. Quantitative Fluorescence Image Analysis

Images were acquired using a FV-1000 Olympus laser confocal scanning system (Olympus, Tokyo, Japan) and Leica Thunder imager 3D live cell and Leica SP8 LIGHTNING Confocal Microscope imaging systems (Leica Microsystems srl, Milan, Italy). Images were acquired under non-saturating conditions (pixel fluorescence below 255 arbitrary units) and analyzed with Fiji ImageJ v.1.52p software. To determine the amount of total PrP, the PrP fluorescence density was measured for the entire cell area excluding the nucleus as a corrected total cell fluorescence (CTCFcell) = integrated density—(area of selected cell × mean background fluorescence) [35]. To determine the level of PrP on the plasma membrane, the CTCF of PrP on the plasma membrane was selected (CTCFpm) and a CTCFpm/CTCFcell ratio was calculated. To determine the amount of PrP in the Golgi apparatus, the CTCF of the PrP fluorescence was measured for the Golgi area of the cell identified by GM130 immunostaining (CTCFgolgi) and for the entire area of that cell excluding the Golgi (CTCFcyt) and the CTCFgolgi/CTCFcyt ratio was calculated. Data are expressed as fold changes relative to control.

### 2.7. Cell Transfection

WT and CaN-KO astrocytes were plated onto 13 mm glass coverslips in 24-well plates (3 × 10^4^ cells/well). Then, 24 h after plating, cells were transfected with plasmids, eGFP PrP or eGFP FFI, in a 1:1 ratio using Lipofectamine 2000 (Thermo Fisher Scientific, Milan, Italy) in Optimem (11058-021; Gibco, Thermo Fisher Scientific, Milan, Italy). The transfection medium complete DMEM was replaced after 12 h, and 48 h after transfection, the cells were washed with PBS and fixed in 4% formaldehyde (Sigma, Milan, Italy). Constructs: the PrP-EGFP constructs eGFP PrP and eGFP FFI, expressing, respectively, mouse PrP (WT-PrP) and mouse D177N/M128 mutant PrP (FFI-PrP), were generated by inserting a monomerized version of EGFP containing a GS linker (GGGGS, repeated four times) at its 3′ end, after codon 34 of 3F4-tagged mouse PrP [46].

### 2.8. Western Blotting

Astroglial cultures were lysed with 100 µL of lysis buffer (50 mM Tris-HCl (pH 7.4), sodium dodecyl sulphate (SDS) 0.5%, 5 mM EDTA), 10 µL of protease inhibitor cocktail (PIC, Millipore, MERK Life Sciences srl, Milan, Italy, Cat. 539133) and phosphatase inhibitor cocktail (Thermo Fisher Scientific, Milan, Italy, Cat. 78428) and collected in a 1.5 mL tube. Lysates were boiled at 96 °C for 5 min and then quantified with a QuantiPro BCA Assay Kit (Sigma, MERK Life Sciences srl, Milan, Italy, Cat. SLBF3463). Then, 40 µg of proteins were mixed with the right amount of Laemmli Sample Buffer 4X (Bio-Rad, Hercules, CA, USA) and boiled. Samples were loaded on a 12% polyacrylamide–sodium dodecyl sulphate gel for SDS-PAGE. Proteins were transferred onto nitrocellulose membrane using Mini Transfer Packs or Midi Transfer Packs with Trans-Blot^®^ Turbo ^TM^ (Bio-Rad, Hercules, CA, USA), according to the manufacturer’s instructions (Bio-Rad, Hercules, CA, USA). The membranes were blocked in 5% skim milk (Sigma, MERK Life Sciences srl, Milan, Italy, Cat. 70166) for 45′ at room temperature. Subsequently, membranes were incubated with indicated primary antibody overnight at 4 °C. The primary antibodies used were anti-mouse monoclonal antibodies 12B2, 94B4; anti-β-Actin was used to normalize protein loading (listed in Table 2). Goat anti-mouse IgG (H + L) horseradish peroxidase-conjugated secondary antibody (1:5000; Cat. 170-6516, Bio-Rad, Hercules, California, USA) and Goat anti-mouse IgG (H + L) horseradish peroxidase-conjugated secondary antibody (1:5000; Cat. 170-6515, Bio-Rad) were used. Detection was carried out with SuperSignal^TM^ West Pico/femto PLUS Chemiluminescent Substrate (Thermo Scientific, Milan, Italy), based on the chemiluminescence of luminol and developed using the ChemiDoc^TM^ Imaging System (Bio-Rad, Hercules, CA, USA).

### 2.9. Preparation of Synaptosomes and Astrocyte Sub-Cellular Fractionation

Synaptosomal fractions were isolated by differential centrifugation using the standard protocol [47]. Briefly, mice were sacrificed followed by decapitation. The brains were rapidly removed and placed into ice-cold homogenization buffer containing 50 mM MOPS, pH 7.4, 320 mM sucrose, 0.2 mM DTT, 100 mM KCl, 0.5 mM MgCl2, 0.01 mM EDTA, 1 mM EGTA, protease inhibitor cocktails (PIC, Millipore, Cat. 539133) and phosphatase inhibitor cocktails (Thermo Fisher Scientific, Milan, Italy, Cat. 78428). All subsequent steps were performed at 4 °C. The hippocampi were microdissected and homogenized in 1:10 *w*/*v* homogenization buffer with 12 strokes in a Teflon glass Douncer. The homogenates were centrifuged for 10 min at 800× *g* followed by centrifugation of the supernatant at 9200× *g* for 15 min. The resulting p pellet, representing the crude synaptosomal fraction, was solubilized in lysis buffer [37].

Astrocyte primary cultures were subjected to the corresponding fractionation to obtain subcellular PrP^C^ distribution, starting from an 80% confluent 100 mm dish of P2 astrocytes, Crt and CaN KO. Cells were lysated in homogenization buffer with 12 strokes in a Teflon glass Douncer. The total lysates (t) were centrifuged for 10 min at 800× *g*, obtaining the post-nuclear supernatant fraction, called s1. The s1 fraction was centrifugated at 9200× *g* for 15 min, obtaining s2, the soluble fraction, and p, the membrane fraction. The resulting p pellet was solubilized in lysis buffer [35,37,48].

### 2.10. Deglycosylation Assay

PrP^C^ was de-glycosylated by incubating cell lysates with PNGase F (Sigma, MERK Life Sciences srl, Milan, Italy, Cat. P7367) according to the manufacturer’s instructions. Samples, treated and not treated with PNGase F, were analysed by western blotting.

### 2.11. Total RNA Extraction and Real-Time PCR

Total mRNA was extracted from 1.0 × 10^6^ cells using TRIzol Lysis Reagent (Invitrogen, Thermo Fisher Scientific, Milan, Italy, Cat. 15596026) according to the manufacturer’s instruction. The first strand of cDNA was synthesized from 0.5–1 µg of total RNA using Im-Prom-II system (Promega, Madison, WI, USA, Cat. A3800). Real-Time PCR was performed using iTaq qPCR master mix, according to the manufacturer’s instructions (Bio-Rad, Hercules, CA, USA, Cat. 1725124), in a SFX96 real-time system (Bio-Rad, Hercules, CA, USA). To normalize raw real-time PCR data, an S18 ribosomal subunit was used. Primers used were listed in Table 3. Data are expressed as delta-C (t) of the gene of interest to S18, allowing appreciation of single gene expression levels.

### 2.12. Proteomic Analysis

Ctr and CaN-KO astrocytes cells were collected, washed and digested with trypsin. Then, 100 µg of protein in 25 µL of 100 mM NH_4_HCO_3_ was reduced with 2.5 μL of 200 mM DTT (Sigma, MERK Life Sciences srl, Milan, Italy) at 90 °C for 20 min and alkylated with 10 μL 200 mM iodoacetamide (Sigma, MERK Life Sciences srl, Milan, Italy, Cat. I5161) for 1 h at RT protected from light. Any excess of iodoacetamide was removed by the addition of 200 mM DTT. The samples were then digested with 5 μg of trypsin (Promega, Madison, WI, USA, Sequence Grade). After an overnight (ON) incubation at 37 °C, 2 μL of neat formic acid was added to stop trypsin activity and the digested samples were dried by speed vacuum [49]. The peptide digests were desalted on a Discovery^®^ DSC-18 solid phase extraction (SPE) 96-well plate (25 mg/well) (Sigma-Aldrich Inc., St. Louis, MO, USA), as reported elsewhere [50].

LC–MS/MS analyses were performed using a micro-LC Eksigent Technologies (Dublin, OH, USA) system with a stationary phase of a Halo Fused C18 column (0.5 × 100 mm, 2.7 μm; Eksigent Technologies, Dublin, OH, USA). The injection volume was 4.0 μL and the oven temperature was set at 40 °C. The mobile phase was a mixture of 0.1% (*v*/*v*) formic acid in water (A) and 0.1% (*v*/*v*) formic acid in acetonitrile (B), eluting at a flow rate of 15.0 μL/min at increasing concentrations of B from 2–40% in 30 min. The LC system was interfaced with a 5600+ TripleTOF system (AB Sciex, Vaughan, ON, Canada) equipped with a DuoSpray Ion Source. Samples were subjected to the traditional data-dependent acquisition (DDA), as previously described [51]. The MS data were acquired with Analyst TF 1.7 (SCIEX, Vaughan, ON, Canada). Three instrumental replicates for each sample were subjected to the DIA analysis [52]. The MS files were searched using the software Mascot v.2.4 (Matrix Science Inc., Boston, MA, USA) using trypsin as enzyme, with 2 missed cleavages, and a search tolerance of 50 ppm was specified for the peptide mass tolerance and 0.1 Da for the MS/MS tolerance; charges of the peptides to search for were set to 2+, 3+ and 4+, and the search was set on monoisotopic mass and FDR at 1%. The instrument was set to ESI-QUAD-TOF, and the following modifications were specified for the search: carbamidomethyl cysteines as fixed modification and oxidized methionine as variable modification. The UniProt/Swiss-Prot reviewed database containing mouse proteins (version 12/10/2018, containing 25,137 sequence entries) was used.

### 2.13. Statistical Analysis

The statistical analysis was performed and related graphical representations were produced using GraphPad Prism v.7. A two-tailed unpaired Student’s *t*-test or one-way Anova test were used. Differences were considered significant at *p* < 0.05.

## 3. Results

### 3.1. CaN KO and FK506 Treatment Reduced PrP^C^ Expression in Mouse Hippocampal Astrocytes

**Figure 1:**
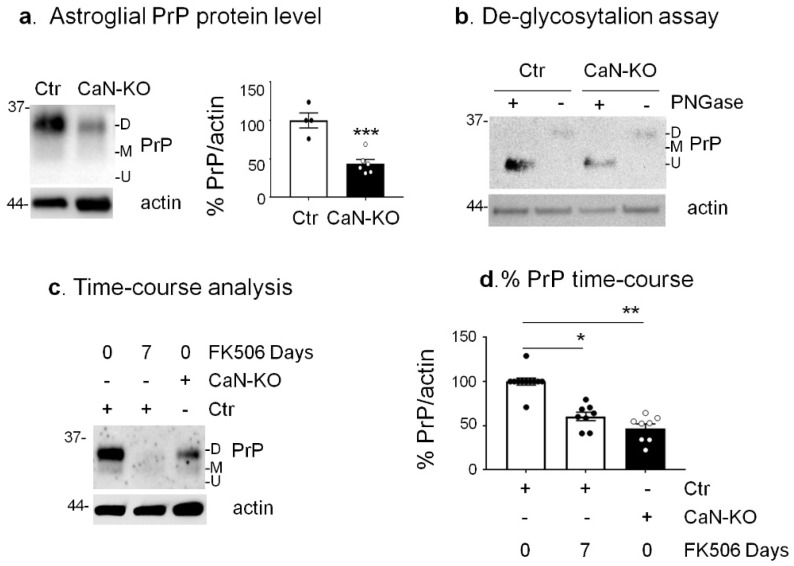
Analysis of PrP^C^ expression in Ctr, FK506-treated Ctr and CaN-KO primary hippocampal astrocytes. (**a**) Expression levels of PrP^C^ in Ctr and CaN-KO astrocytes. WB analysis of protein extracts with anti-PrP^C^ 12B2 and anti-actin antibodies and quantification of actin-normalized PrP^C^ levels expressed as percentages of Ctr. Data are the means ± SEM of Ctr (*n* = 4) and KO (*n* = 6) replicate astrocyte cultures. *** *p* < 0.001 by unpaired *t*-test. (**b**) Deglycosylation assay in Ctr and CaN-KO astroglial lysates. Ctr and CaN-KO astroglial lysates were treated with PNGase F and analysed by WB with anti-PrP 12B2 and anti-actin antibodies. (**c**) Time-course analysis with FK506 in Ctr and CaN-KO astrocytes. Ctr astrocytes were left untreated or treated with FK506 200 nM for 7 days and analyzed by WB with anti-PrP 12B2 and anti-actin antibodies, along with CaN-KO astrocyte lysates. (**d**) Quantification of actin-normalized PrP^C^ levels in WB like the one shown in (**c**). Data are the means ± SEM, (*n* = 6–11); * *p* < 0.05, ** *p* < 0.01 vs. Ctr by one-way Anova, multiple comparison Kruskal–Wallis test.

Previously, we have shown that CaN in astrocytes regulates the expression of plasma membrane proteins, e.g., glial high-affinity glutamate–aspartate transporter (GLAST) [35]. Given the emerging role of astrocytic PrP^C^ [8,9], we have investigated whether PrP^C^ could be regulated by CaN. First, we evaluated the protein expression levels of PrP^C^ in primary cultures of hippocampal astrocytes from control and astrocyte-specific CaN-KO (ACN-Ctr and ACN-KO) mice, hereafter referred to as Ctr and CaN-KO astrocytes. We found that CaN-KO astrocytes had a reduced expression of PrP^C^ compared to Ctr astrocytes by about 50% (Figure 1a). We observed that, in total cell lysates, the major PrP^C^ signal resulted from diglycosylated bands, as shown in Figure 1a, while the mono-glycosylated or non-glycosylated forms were less expressed in both Ctr and CaN-KO astrocytes. As expected, deglycosylation with PNGaseF produced a single deglycosylated band of approximately 23 KDa in both Ctr and CaN-KO astrocytes (Figure 1b).

Next, we decided to verify if the pharmacological inhibition of CaN reduced PrP^C^ levels like in astrocytes with genetic CaN ablation. Treatment of Ctr astrocytes with FK506 (200 nM for 7 days) resulted in a significant decrease of PrP^C^ of about 45% (Figure 1c,d), confirming that the reduction of endogenous PrP^C^ proteins in CaN-KO astrocytes was due to the inhibition of CaN activity.

### 3.2. Total and Membrane PrP^C^ Downregulation

**Figure 2:**
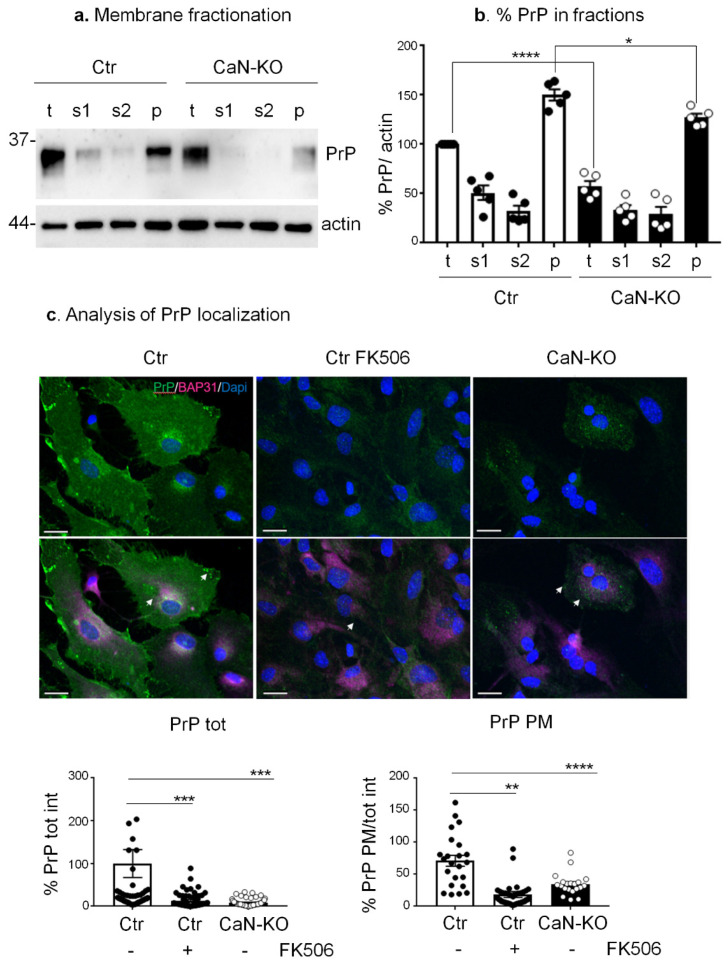
Analysis of PrP^C^ localization in Ctr, FK506-treated Ctr and CaN-KO primary hippocampal astrocytes. (**a**) Fractionation of Ctr and CaN-KO astrocytic lysates by centrifugation and evaluation of PrP^C^ relative abundance by WB with anti-PrP in t (total cell lysates), s1 (post nuclear supernatant), s2 (soluble fraction), p (membrane fraction) fractions. (**b**) Quantification of membrane fractionation is expressed as means ± SEM, *n* = 5; **** *p* < 0.0001, * *p* < 0.05 by one-way Anova, Sidak’s multiple comparison. (**c**) Immunofluoresce analysis on Ctr, FK506-treated Ctr and CaN-KO astrocytes with anti-PrP (green), anti-BAP31 (pink) antibodies and reacted with DAPI to stain the nuclei (blue). Confocal microscope analysis, scale bar 20 µm. Quantification of total PrP (PrP tot) or plasma membrane PrP (PrP PM) fluorescence density expressed as percentages of Ctr from Ctr *n* = 54 cells, FK506-treated Ctr *n* = 67 and CaN-KO *n* = 70 cells, from four to five replicates, ** *p* < 0.01, *** *p* < 0.001 and **** *p* < 0.0001.

Expression of PrP^C^ at the plasma membrane has been specifically associated with its ability to be converted to misfolded PrP and to the development of pathology, both in vitro and in vivo [53,54]. Therefore, our next question was whether the reduction of PrP^C^ in CaN-KO astrocytes occurs specifically at the plasma membrane level. For this, we have fractionated cell lysates to isolate soluble and membrane proteins by centrifugation, as has previously been performed on tissue [37]. Briefly, the total cell lysate was centrifuged to obtain a post-nuclear supernatant (s1), which subsequently was separated into the soluble fraction (s2) and the membrane fraction (p). As shown in Figure 2a,b, in total lysates the PrP^C^ signal was reduced in CaN-KO astrocytes compared to Ctr. The sub-cellular fractionation procedure showed an enrichment of PrP^C^ in fraction p both in Ctr and CaN-KO compared to total lysate, s1 or s2, while a reduction of PrP levels, specifically in the p fraction of CaN-KO astrocytes, was observed compared to the p fraction from Ctr cells, indicating the reduction of membrane-associated PrP^C^. To strengthen the results, we immunostained CaN-KO (or FK506-treated) astrocytes with anti-PrP antibody and quantified the ratio of membrane and total PrP^C^. As shown in Figure 2c, both genetic and pharmacological ablation of CaN in astrocytes resulted in a marked reduction of the total and of the membrane PrP^C^ signal compared to Ctr.

**Figure 3:**
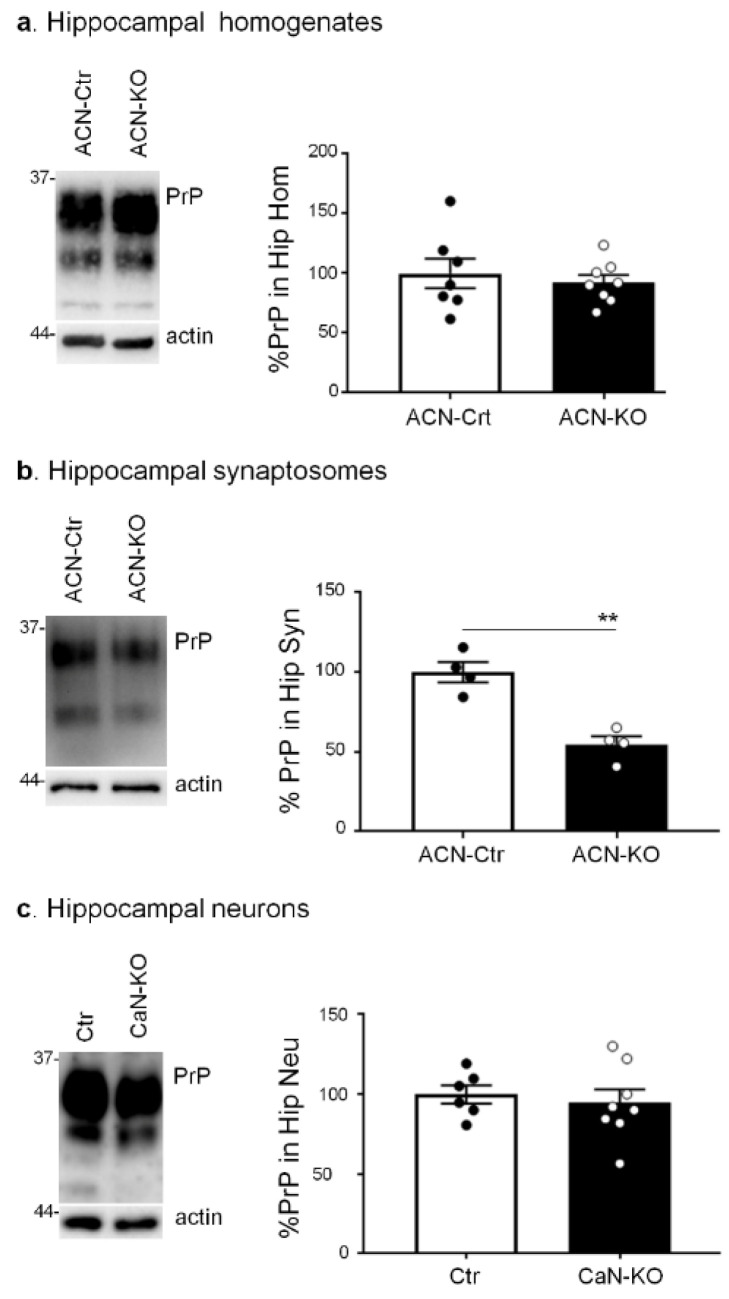
Analysis of PrP^C^ expression in hippocampal tissues from ACN-Ctr and ACN-KO mice at one month of age. (**a**) WB analysis with anti-PrP and anti-actin antibodies of hippocampal homogenates from ACN-Ctr and ACN-KO mice. Quantification of the actin-normalized PrP signal expressed as percentage of Ctr. Data are the means ± SEM, ACN-Ctr *n* = 7, ACN-KO *n* = 8, unpaired *t*-test, ns. (**b**) Synaptosomal fractions from hippocampi of ACN-Ctr and ACN-KO mice were analyzed by WB with anti-PrP and anti-actin antibodies. Quantification of the actin-normalized PrP signal expressed as percentage of Ctr. Data are the means ± SEM, ACN-Ctr *n* = 4, ACN-KO *n* = 4, unpaired *t*-test, ** *p* = 0.0015. (**c**) WB analysis with anti-PrP and anti-actin antibodies of hippocampal neurons from ACN-Ctr (Ctr) and ACN-KO (CaN-KO) mice. Quantification of the actin-normalized PrP signal expressed as percentages of Ctr. Data are the means ± SEM, Ctr *n* = 6, CaN-KO *n* = 8, unpaired *t*-test, ns.

Next, we investigated whether CaN-dependent PrP^C^ reduction could be detected in vivo in total hippocampal homogenates from ACN-Ctr and ACN-KO mice at 1 month of age, i.e., the age-point when mice develop deregulation of neuronal excitability and protein expression [36,37]. As reported in Figure 3a, an equal level of PrP^C^ was expressed in whole-tissue hippocampal homogenates from ACN-Ctr and ACN-KO mice, which can be attributed to higher PrP^C^ neuronal protein expression, which was in apparent contradiction with in vitro data. One of the morpho-functional units of astrocytes is represented by the fine astrocytic processes surrounding synapses, peri-synaptic processes, in which ion and metabolite transporters and other proteins are enriched and which may represent the site of localization of PrP^C^ in astrocytes in vivo. To test this hypothesis, we prepared synaptosomal fractions from ACN-Ctr and ACN-KO hippocampi, using a previously reported protocol which preserves and enriches perisynaptic astrocytic processes [35,37]. Indeed, we found a significant reduction of PrP^C^ in the synaptosomal preparation from hippocampi of ACN-KO compared to ACN-Crt mice (Figure 3b). To rule out the neuronal PrP^C^ contribution to the reduction of PrP levels in synaptosomes, we assessed PrP^C^ expression in primary hippocampal neurons from ACN-Ctr and ACN-KO mice. We observed that Ctr and CaN-KO neurons expressed equal levels of PrP^C^ (Figure 3c), supporting the view that the reduction of PrP^C^ in vivo occurs in astrocytes but not in neurons.

### 3.3. CaN Ablation-Induced PrP^C^ Downregulation Is not due to Alterations of Gene Expression or Protein Degradation

In many cell types, including astrocytes, CaN is known to control expression, first of all, through regulation of gene transcription [10,20,55]. Therefore, we checked if CaN deletion or pharmacological inhibition could alter *Prnp* gene transcription.

**Figure 4:**
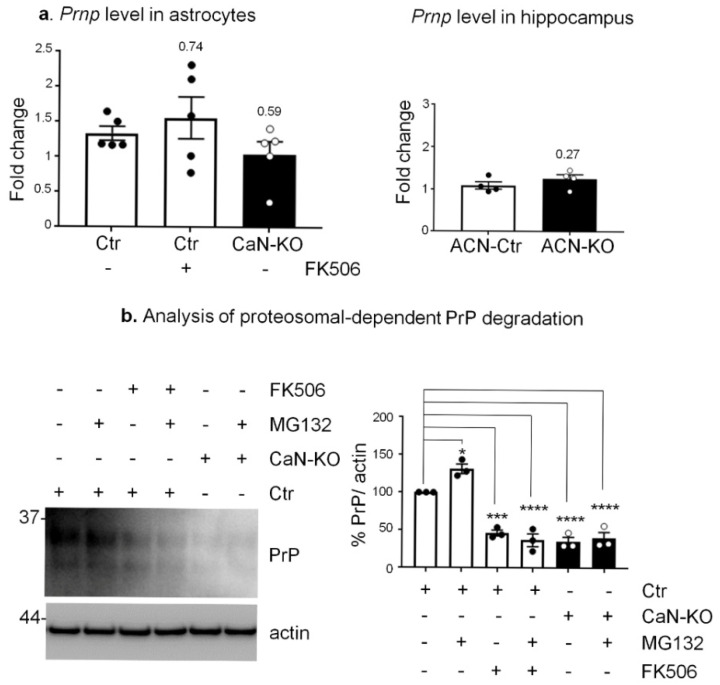
Real-time PCR, proteasomal degradation analysis of Ctr, FK506-treated Ctr and CaN-KO astrocytes. (**a**) Real-time PCR of *Prnp* on primary astrocytes, Ctr, FK506 (200 nM for 7 days)-treated and CaN-KO. Values represent means ± SEM ∆C(t) of gene/S18 of five independent experiments for each condition. (**b**) WB analysis of PrP^C^ and actin, protein degradation in hippocampal astrocytes from Ctr, FK506 (200 nM for 7 days)-treated and CaN-KO. Where indicated, MG132 was added 3 h before lysis. Data are expressed as means ± SEM, three independent cultures were used, one-way Anova, multiple comparison, * *p* < 0.05, *** *p* < 0.001 and **** *p* < 0.0001.

**Figure 5:**
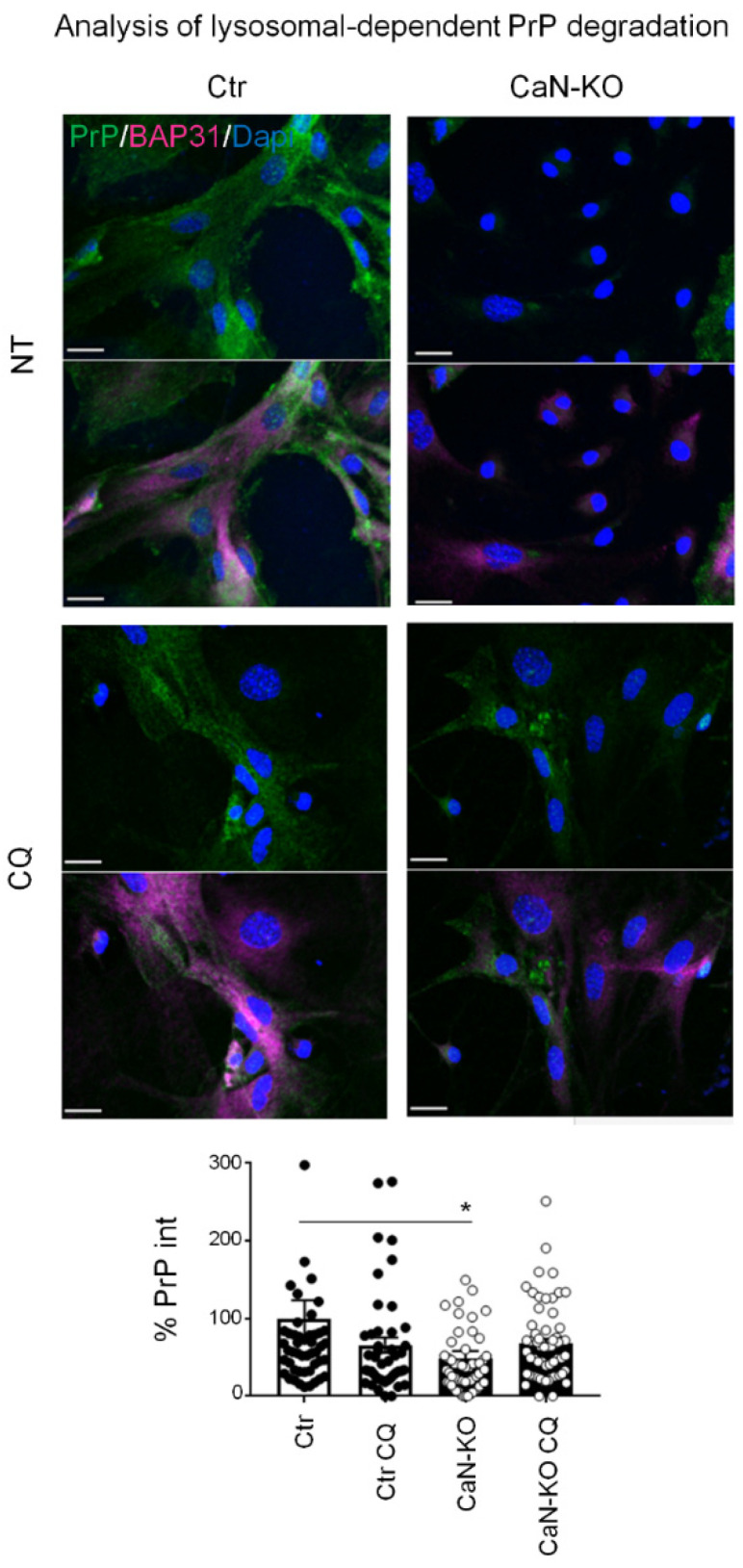
Analysis of lysosomal-dependent PrP^C^ degradation. Ctr and CaN-KO astrocytes were untreated or treated with chloroquine (CQ) before immunofluorescence with anti PrP (green), BAP31 (pink) antibodies and reacted with DAPI to stain the nuclei (blue). Confocal microscope analysis, scale bar 20 µm. Quantification of total PrP^C^ fluorescence density expressed as percentage of Ctr. Data are the means ± SEM of Ctr *n* = 49 cells, Ctr CQ *n* = 46 and KO *n* = 58 cells; KO CQ = 62, from three to seven coverslips from three independent experiments, * *p* < 0.05.

As shown in Figure 4a, *Prnp* mRNA levels were unchanged in: (i) Ctr astrocytes treated for 1 week with FK506 vs. Ctr astrocytes; (ii) cultured CaN-KO vs. Ctr astrocytes; and (iii) hippocampal tissues from ACN-KO vs. ACN-Ctr mice, ruling out the possibility of transcriptional regulation. Next, we investigated if CaN-dependent PrP^C^ reduction was due to alteration of proteasomal or lysosomal degradation [28,35], since it has been shown that in neurons lysosomes contribute to degradation of PrP^C^ [35]. For this, we treated astrocytes either with MG132, a specific proteosomal inhibitor, or with CQ, an anti-malaric agent and a commonly used inhibitor of lysosomal degradation. As for proteasomal degradation, PrP expression increased when Ctr astrocytes were treated with MG132, suggesting an accumulation of synthetized but not degraded PrP^C^. However, MG132 failed to rescue the downregulated PrP^C^ expression in CaN-KO astrocytes or in astrocytes treated with FK506 (Figure 4b). CQ treatment did not alter significatively the levels of PrP^C^ expression in control astrocytes and failed to rescue decreased PrP^C^ expression in CaN-KO astrocytes (Figure 5). Altogether, these data suggest that the CaN ablation-dependent reduction of PrP^C^ expression in astrocytes is neither due to altered transcription nor due to augmented degradation of the protein.

### 3.4. Reduction of PrP^C^ Expression in CaN-KO Astrocytes Results from Deregulation of Global Protein Synthesis

**Figure 6:**
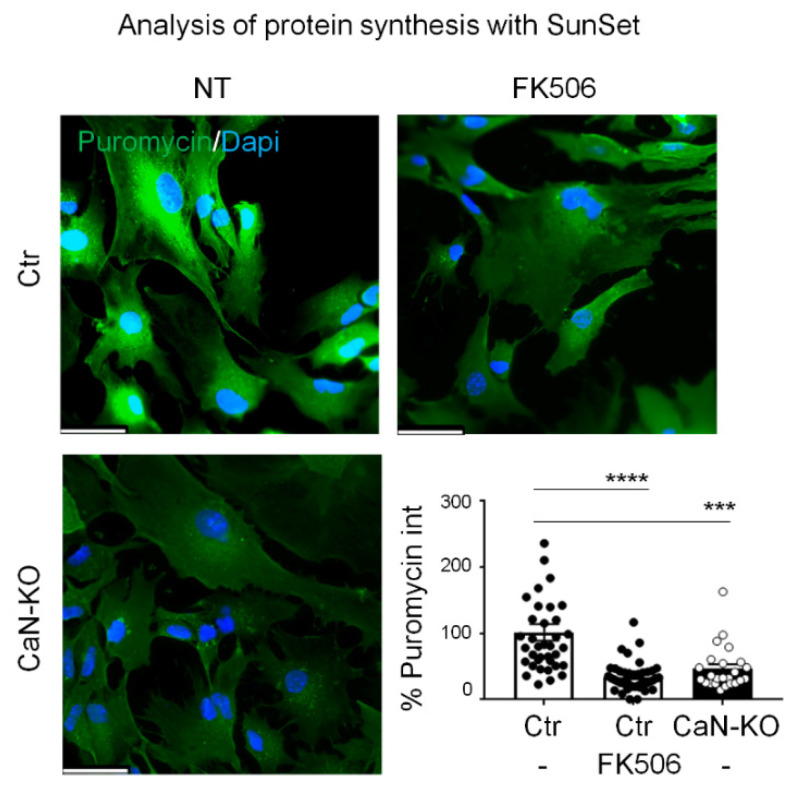
Protein synthesis analysis in Ctr, FK506-treated Ctr and CaN-KO astrocytes. Ctr, FK506-treated Ctr and CaN-KO astrocytes were pulsed with 4 µM puromycin, fixed and analysed by IF with anti-puromycin antibody (green) and reacted with DAPI to stain the nuclei (blue). Images were acquired with a Leica Thunder imager 3D live cell microscope, scale bar 41.6 µm. Data are expressed as means ± SEM of *n* cells Ctr = 37, FK506-treated Ctr = 44, CaN-KO = 24, from three independent experiments. one-way Anova, Dunnett’s multiple comparison analysis, *** *p* < 0.001 and **** *p* < 0.0001.

Recently, we have shown that both genetic and pharmacological ablation of CaN in astrocytes suppresses global protein synthesis [35]. In the absence of transcriptional and post-translational alterations (see above), it is reasonable to suggest that the impairment of protein synthesis alone could account for the reduced PrP^C^ protein expression in CaN-KO and FK506-treated astrocytes. To pursue this hypothesis, first we confirmed, using an immunocytochemical variant of the puromycin incorporation assay [45], that active ribosomes in CaN-KO astrocytes and Ctr astrocytes treated with FK506 incorporated less puromycin, suggesting an impairment of global protein synthesis (Figure 6). Protein synthesis is a complex multistep process in which CaN has been suggested to regulate several steps [56]. To shed light on possible mechanisms of protein synthesis deregulation in CaN-KO astrocytes, we performed shotgun mass spectrometry proteomics followed by bioinformatic analysis.

As shown in Table S1a, 1212 and 823 proteins were identified, respectively, in Ctr and CaN-KO astrocytes. Of these, 609 were commonly expressed by both types of astrocytes, while 603 and 214 were identified only in Ctr or CaN-KO astrocytes, respectively (see also Table S1a,b). The analysis using the DAVID online gene ontology (GO) tool revealed that the most significantly overrepresented GO terms in Ctr astrocytes were related to translation, ribosomes, focal adhesion and components of the extracellular matrix, and the most significantly overrepresented KEGG pathway was Ribosome (Table S1b). GO terms, overrepresented in CaN-KO astrocytes, were related to RNA splicing, RNA binding, focal adhesion and the extracellular matrix (Table S1c). Overall, this analysis suggests that ribosome-mediated translation may be specifically impaired in CaN-KO astrocytes, which links the downregulation of PrP^C^ in CaN-KO astrocytes to protein synthesis.

### 3.5. FK506 and CaN-KO Reduced the Expression and Plasma Membrane Localization of WT and Mutant PrP Associated with Human Inherited Prion Diseases

Since prion diseases include genetic variants caused by mutations in the PrP gene, including the PrP_D177N_ mutation associated with FFI human disease [31,46,57,58], it was of interest to investigate whether the effect of CaN KO in astrocytes differs between wildtype PrP^C^ and the mutant variant.

**Figure 7:**
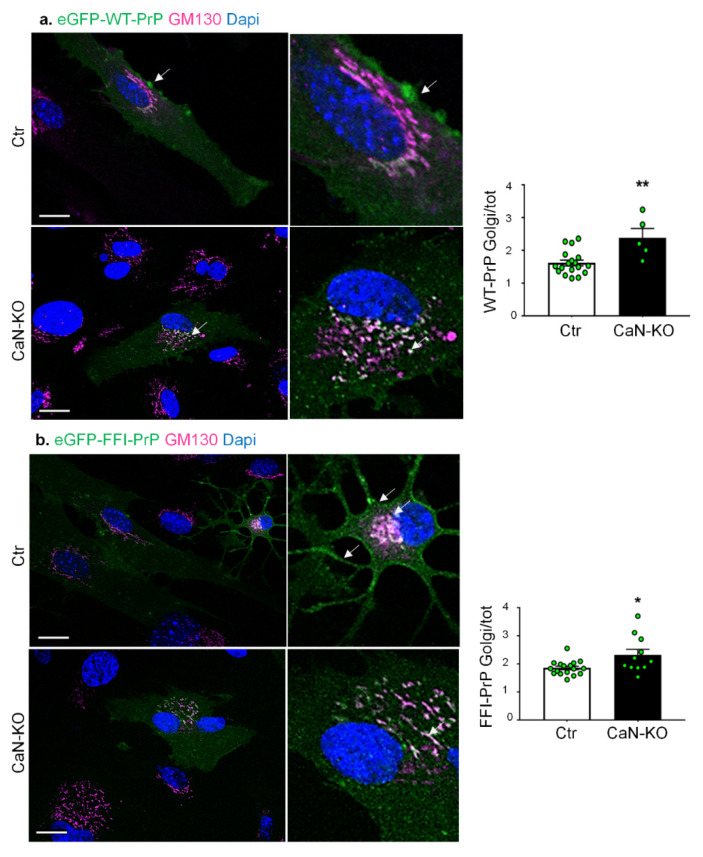
Analysis of WT and D177N PrP transient expression in Ctr and CaN-KO astrocytes. Ctr and CaN-KO astrocytes were transfected with eGFP-WT PrP (**a**) and eGFP-D177N PrP (**b**) fusion proteins (green), immunostained with an anti-GM130 (pink) to mark the Golgi apparatus and reacted with DAPI to stain the nuclei (blue). Images were acquired with a confocal microscope, scale bar 20 µm. PrP fluorescent density in the Golgi apparatus is expressed as mean ± SEM, from four independent coverslips, * *p* < 0.05 and ** *p* < 0.01.

For this, we transiently transfected Ctr and CaN-KO astrocytes with eGFP-tagged PrP_WT_ or PrP_D177N_ [57]. In neurons, it has been shown that overexpressed PrP proteins, including mutant variants, are partially retained in the Golgi apparatus compartment. Therefore, using confocal microscopy, we quantified the ratio between plasma membrane-localized PrP and PrP localized in the Golgi compartment. The analysis showed that both PrP_WT_ and PrP_D177N_ were expressed significantly less at the plasma membrane of CaN-KO as compared with Ctr astrocytes, suggesting that the effect of CaN KO is maintained also in mutant variants of PrP (Figure 7).

## 4. Discussion

Previously, we have reported that CaN controls the expression of membrane proteins, e.g., GLAST, through a dynamic regulation of protein synthesis and degradation [35]. The aim of this study, therefore, was to see if CaN could also be involved in the control of PrP^C^ expression. The main results of this study are as follows: (i) genetic ablation of CaN from astrocytes as well as chronic treatment of cultured astrocytes with a CaN inhibitor reduced PrP^C^ expression in astrocytes but not in neurons; (ii) the reduction of PrP^C^ in astrocytes was due to the impairment of protein synthesis machinery but not due to alterations of transcription or protein degradation; (iii) the reduction of PrP^C^ protein expression in astrocytes is associated with the reduction of its presence on the plasma membrane, which is true for both WT PrP^C^ and mutant FFI-related PrP.

In spite of significant progress in the field, the downregulation of PrP^C^ remains one of the most promising approaches to mitigate the conversion of PrP^C^ in PrP^Sc^ and the burden of prion disease [38,39,41,42,43,59]. However, it has been suggested that PrP^C^ regulates many cellular functions, including neuronal excitability, differentiation, ion homeostasis and mitochondrial functions and that it also plays a role in immune cells [60]. In neurons, PrP^C^ has been proposed to serve as a molecular scaffold for the transduction of signals across the plasma membrane [61,62]. We and other groups have shown that endogenous PrP^C^ regulates neuronal calcium signalling, in particular, store-operated calcium entry and glutamate-induced mitochondrial accumulation of Ca^2+^ [63,64,65,66]. Therefore, systemic downregulation of PrP^C^ may result in harmful drawbacks for the cell due to ablation of its physiological activity. In this framework, the data presented here, in correlation with reports on the role of astrocytic PrP^C^ in spreading the prion pathology, suggest that astrocytic CaN may represent a valuable strategy to counteract prion diseases.

Inhibitors of CaN have already been suggested to mitigate neuropathology in models of Alzheimer’s disease, strokes and, importantly, in mouse models of acquired prion disease [27,28,29,67,68,69,70,71,72]. In the context of neuropathology, overactivation and/or overexpression of CaN, specifically in astrocytes, has been associated mainly with reactive gliosis and neuroinflammation. Thus, the role of the CaN-mediated activation of transcriptional activity of NFAT and its association with neuroinflammation has been largely discussed in the context of Alzheimer’s disease [11,20,73,74], highlighting the benefits of astrocyte-specific targeting.

In this context, it is important to emphasize that the CaN ablation-mediated downregulation of PrP^C^ is not related to pro-inflammatory effects of CaN or to transcriptional activation. Instead, we suggest that CaN, through protein dephosphorylation, regulates translational machinery, as has been already proposed [56,75,76]. Previously, we have shown that, in resting conditions, astrocytic CaN does not regulate gene transcription, acting through dephosphorylation of target proteins, and that one of the target processes may be the assembly of ribosomal complexes and initiation of translation [36,37,56,75,76]. One of the results of such deregulation is the impairment of the expression of ribosomal proteins in CaN-KO astrocytes, which is supported by our present proteomics analysis (Table S1). We have also shown that CaN ablation regulates expression of the astrocytic glutamate transporter, GLAST, at a post-transcriptional level through a disequilibrium between protein synthesis and degradation [35]. Both genetic and chronic pharmacological CaN inhibition resulted in upregulation of GLAST, an opposite effect to that found here for PrP^C^. This suggests that the deregulation of protein expression upon CaN inhibition is protein-specific and may result in either upregulation or downregulation of protein expression [37].

In conclusion, our results are in line with the suggestion that anti-CaN treatment may be beneficial in prion diseases. At the same time, the results provide mechanistic insight into astrocyte-specific PrP^C^ regulation, highlighting the possibility of a non-neuronal yet cell-specific approach to reduce the burden of misfolded PrP and mitigate the development of prion diseases.

## Figures and Tables

**Table 1:** Primary antibodies used for IF analysis.

Antibodies	Dilution	House	Cat.
Anti-puromycin	1:200	Millipore	MABE343
12B2	1:400	Central Veterinary Institute	/
Anti-GM130	1:500	Transduction Laboratories	610823
Anti-BAP31	1:200	Proteintech	11200-1-AP

**Table 2:** Primary antibodies used for WB analysis.

Antibodies	Dilution	House	Cat.
94B4	1:1000	Wageningen University & Research	mAbPrP94B4
12B2	1:500	Central Veterinary Institute	/
Anti-β-Actin	1:800	Sigma	A1978

**Table 3:** Oligonucleotide primers used for real-time PCR.

Protein	Gene	Forward/Reverse	Accession No.
S18	*Rps18*	TGCGAGTACTCAACACCAACA CTGCTTTCCTCAACACCACA	NM_011296
PrP	*Prnp*	GAACCATTTCAACCGAGCTGA TAGTCACAAAGAGGGCCAGC	NM_011170.3

## Data Availability

Not applicable.

## Supplementary Materials

The following are available online at https://www.mdpi.com/article/10.3390/cells11040609/s1, Table S1: Proteomic analysis on Ctr and CaN-KO astrocytes.
